# Functional assessments used by occupational therapists with older adults at risk of activity and participation limitations: a systematic review and evaluation of measurement properties

**DOI:** 10.1186/2046-4053-1-45

**Published:** 2012-10-15

**Authors:** Kylie Wales, Lindy Clemson, Natasha A Lannin, Ian D Cameron

**Affiliations:** 1 Ageing Work and Health Research Unit and The Centre for Excellence in Population Ageing Research, Faculty of Health Sciences, The University of Sydney, PO BOX 170, Lidcombe, NSW, 2141, Australia; 2 Occupational Therapy Department, Alfred Health, and Faculty of Health Sciences, Alfred Clinical School, La Trobe University, The Alfred, 55 Commercial Road, Prahran, VIC, 3181, Australia; 3 Rehabilitation Studies Unit, Sydney Medical School, The University of Sydney, PO BOX 6, Sydney, NSW, 2112, Australia

**Keywords:** Occupational therapy, Function, Older adults, Assessment, Measurement properties

## Abstract

**Background:**

Older adults experience activity and participation limitations that are associated with ageing. Activity and participation limitations affect an older adult’s ability to engage in meaningful daily activities and valued life roles. Occupational therapists provide interventions to reduce such limitations and monitor client change to ensure that interventions are effective. Client change should be measured through the use of valid and reliable assessments. Yet occupational therapists can favour the use of non-standardised assessments leading to inaccurate reflections of client change and difficulties in comparing the effectiveness of interventions. A number of reasons have been suggested as to why therapists may favour non-standardised assessments, including a lack of knowledge (of assessments and their properties) and lack of skill.

**Methods/design:**

This paper describes the systematic review protocol that will be used to identify functional assessments used in randomised trials of occupational therapy interventions for older adults (≥70 years of age). Interventions will focus on enhancing functional independence for either older adults transitioning from hospital to home, or community dwelling older adults. We will search Medline, EBSCO and OTseeker using a pre-determined search strategy to identify Functional assessments. These assessments will be recorded and, in phase two, their measurement properties analysed.

**Discussion:**

This protocol provides a comprehensive guideline for conducting the proposed systematic review. The results of this systematic review will provide a thorough and unbiased identification and evaluation of measurement properties of functional assessment tools used in randomised trials to evaluate occupational therapy intervention. This information can be used to determine which assessment has superior measurement properties and will inform occupational therapy practice.

## Background

Ageing results in an increased risk of chronic disease and disability, all of which contributes to the demand for acute and chronic healthcare services [1]. In Australia, the largest prevalence of disability is seen in groups aged 70 years and older, with the highest number of people with a disability seen in the 90 years and over age group (70.3%) [2]. Disability is comprised of three areas of functioning: 1) impairment, 2) activity limitations and 3) participation restrictions [3]. Older adults with a disability often require some form of support to live independently [4]. As such, reducing functional limitations experienced by older adults is an essential part of healthcare services.

Occupational therapists have long identified the link between engagement in meaningful daily activities and health and well-being [5]. As such, therapists aim to reduce functional limitations by providing interventions based on activity and participation. Current research has established occupational therapy’s role with older adults in enhancing functional independence in a number of areas for older adults, including community and stroke services, for example, [6-9].

An important component of therapy is the evaluation of the intervention provided [10]. With an increasing demand on the healthcare system, it is imperative that services are demonstrating the effectiveness of interventions [10]. To provide accurate reflections of practice, occupational therapists should use valid and reliable assessments [11,12]. Yet, occupational therapists are favouring the use of non-standardised evaluations [13-15]. With a number of valid and reliable assessments available for use, (for example. the Nottingham Extended Activities of Daily Living Scale [16]), questions must be raised as to why therapists are not routinely using these. Readiness of therapists to use validated assessment tools, skill, time, motivation, self-confidence, lack of support from management, personal values and beliefs, and lack of knowledge have all been suggested as reasons for limited uptake [13,15,17-19].

There is little information regarding which functional assessment should be used by occupational therapists when working with older adults. Reviews of functional assessments from an occupational therapy perspective are available but lack methodical selection of assessments, leading to inaccurate reflections of assessment use. Law and Letts, 1989, conducted a literature search of assessment tools used to predict or evaluate activities of daily living (ADL). Authors reviewed the: 1) purpose of scale, 2) clinical utility, 3) scale construction, 4) standardisation, and 5) reliability and validity of identified assessments [20]. The authors concluded that no new ADL assessments should be created; instead, further research should be conducted to enhance current assessments. The Index of ADL, Barthel Index, the revised level of rehabilitation scale and physical maintenance scale were found to have the highest reliability and validity of those reviewed. The population and setting of interest was not specified by authors, which creates difficulty in translating results to practice. A similarly conducted study by Klein *et al*., 2008, compared 18 functional assessments to the Canadian Model of Occupational Performance to determine whether the assessment measured key occupational therapy perspectives [21]. Again, no information relating to population of interest or setting was provided, and no decisions relating to which assessment should be used in practice were made. Other research includes a literature review of six assessments (Candian Occupational Performance Measure, Assessment of Motor and Process Skills, McMaster Toronto Arthritis, Goal Attainment Scale, Target Complaints and Patient Specific Function) [22]. Authors described the measurement properties of each and concluded the need for occupational therapists to use assessments that are psychometrically sound and reflect practice. Since these tools do not measure the same construct, no definitive conclusions regarding use in practice can be drawn. The proposed review will identify functional assessments used in randomised trials to measure the effectiveness of occupational therapy interventions for at-risk older adults. We expect that reviewing randomised trials will capture a comprehensive selection of functional assessments, and that tools used in these trials would be of higher quality than in-house developed assessments of function that therapists favour in practice. In the second phase of this study, a comprehensive review of measurement properties of each assessment will be completed. The results of this study will provide an objective identification and evaluation of measurement properties of functional assessments used with older adults. Without such comparisons, therapists will continue to use a variety of different assessments which makes benchmarking of practice impossible. Identifying assessments used for measuring functional independence in older adults and their measurement properties will provide therapists with information needed to make informed decisions about the choice of assessment tools for practice.

This protocol outlines the methods to be used to systematically identify functional assessments used by occupational therapists with older adults at-risk of activity and participation limitations. The protocol also describes the process for reviewing the measurement properties of each assessment identified.

### Review questions

The review questions are as follows:

I. Phase one, systematic identification of functional assessments used in randomised trials: What functional assessments are used by occupational therapists to measure the effectiveness of enhancing functional independence for older adults at-risk of activity and participation limitations?

II. Phase two, measurement properties: Of the identified functional assessments, which is psychometrically superior for use with older adults at-risk of activity and participation limitations?

The definition of older adults at risk of functional limitations that will be applied to the review are: persons aged 70 years and over with one or more functional difficulties, who are transitioning from hospital to home or are community dwelling. Functional limitations and difficulties will be defined as limitations in activity performance and participation as described by the International Classification of Functioning (ICF) [3]. People with pre-existing functional difficulties are also at risk of further functional decline [23] and will be included in this review.

### Types of publications/studies

Randomised trials published in peer reviewed literature will be considered for this review. Articles must be published in English.

### Settings and participants

Participants will be adults aged 70 years or older. Studies will be included where 50% or more of participants are 70 years and over. Participants will either need to be transitioning from hospital to home, or be community dwelling, and recipients of any occupational therapy intervention that aims to enhance functional independence. An occupational therapy intervention is one that is designed and/or implemented by an occupational therapist, provided solely or within a team setting. For example, if a rehabilitation team was providing functional intervention to an older adult and the occupational therapist had a component of intervention, this study would be included in the review.

### Types of methods

Randomised trials in which a functional assessment is administered will be included. If the trial is a cross-over trial, both arms may be considered, if relevant.

### Types of outcomes

Assessments will be included that measure activity performance and participation as defined using the ICF [3] will be included. Assessment tools which measure ICF impairment will be excluded.

### Search methods for the identification of studies

The following electronic databases will be searched: 1) Medline, 2) EBSCO, 3) OTseeker. Medical subject headings (MeSH) and text words will be combined in search strategies (Medline search strategy is attached in Additional file 1). Searches will be restricted to studies published in English. Reference lists of included studies will be independently searched by one reviewer to identify additional studies.

## Methods/design

### Screening

After searches have been completed, abstracts will be downloaded into the reference management system EndNote and duplicates removed. Duplicates will also be removed by hand as required. A study will be considered duplicate if the following are common: 1) authors, 2) location and setting, 3) interventions, 4) number of participants and baseline data, and 5) date and duration of study [24].

**Figure 1:**
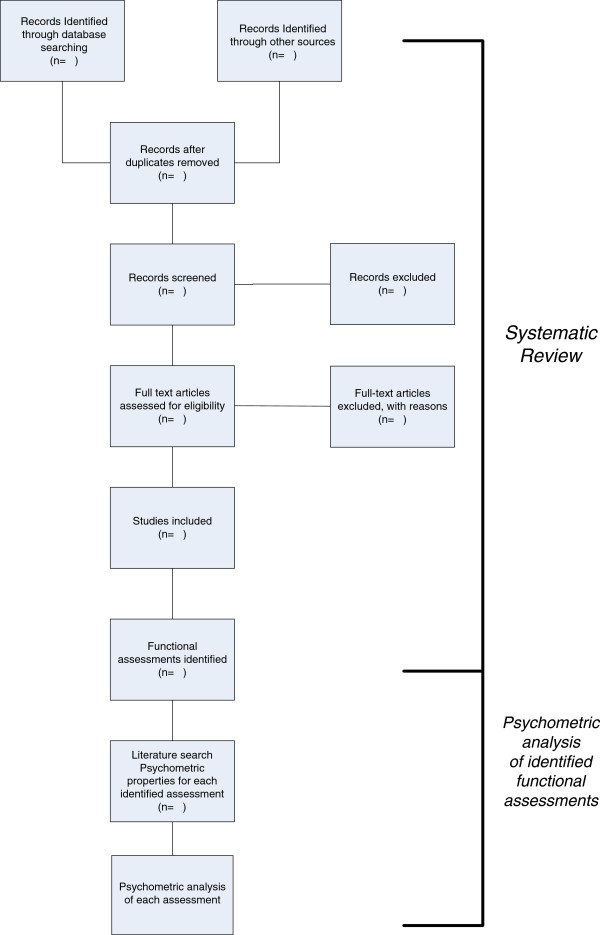
Systematic review process.

Potential studies will be first screened on title, then abstract and finally full manuscript to determine eligibility, see Figure 1. The title and abstract, as needed, of each trial will be screened by one review author. Potential studies, which are not excluded, will be further screened independently by two authors, first on abstract and then, if required, the full manuscript. Differences in opinions regarding trial eligibility will be resolved through discussion and consensus of three authors.

### Data management

We will record and report the details of all studies identified in searches, the number of studies (once duplicates are removed), the number of full text papers obtained and the number and reasons for excluded studies [25].We will manage this data in Endnote.

### Data extraction

Data will be extracted using a standardised data extraction form. This form will record information related to participants, study design, description of intervention, functional assessments used/administered and measurement properties, if described, study inclusion/exclusion criteria and a brief summary of findings.

### Risk of bias assessment

The purpose of this article is to systematically identify functional assessment tools used by occupational therapists and to evaluate the measurement properties of each assessment that is identified. As such, a risk of bias assessment is outside the scope of this review and will not be completed.

### Data analysis

For the first review question, a list of each functional assessment tool identified in the review will be presented.

### Phase two, measurement property evaluation

To identify research relating to measurement evaluation, Medline, EBSCO and Embase will be searched. Contact with initial developers of the assessment will be made where possible. For database searches, the name and known abbreviation of the assessment tool will be used in conjunction with a search strategy based on Terwee *et al*., 2009 (adapted for each database) [26]. The assessment tool’s quality can be affected by the reporting available for that particular tool [27]. By appraising the methodological quality of such articles one can be assured that appropriate conclusions are drawn regarding measurement properties [28]. The Consensus-based standards for the selection of health measurement instruments (COSMIN) 4-point checklist will be used to determine overall methodological quality of each included study [28]. Once methodological quality has been determined, Terwee’s criteria relating to good measurement properties will be applied [27]. Terwee’s criteria provides definitions relating to nine measurement properties (content validity, internal consistency, criterion validity, construct validity, reproducibility, responsiveness, floor and ceiling effects and interpretability), which are considered essential in high quality assessment tools. With the use of Terwee’s quality criteria, decisions can be made regarding which functional assessment tool is the highest quality [27].

The COSMIN checklist is designed as a modular tool and, therefore, only criteria reported in the study under review will be extracted. Data extracted according to the COSMIN criteria includes information reported on: 1) internal consistency, 2) reliability (test-retest reliability, inter-rater reliability and intra-reliability), 3) measurement error, 4) content validity (including face validity), 5) structural validity, 6) hypotheses testing, 7) cross cultural validity, 8) criterion validity, 9) responsiveness, 10) interpretability, 11) item response theory (for tools which have used this technique) and 12) generalisability [28]. Additional information will be extracted in accordance with Terwee’s criteria, including construct validity, reproducibility, and floor and ceiling effect [27]. Feasibility information, as developed by Steiner and adapted by Zwakhalen, will also be collected [29,30]. This analysis will be conducted by two reviewers, in line with COSMIN recommendations.

## Discussion

This systematic review will identify which functional assessments are used by occupational therapists when working with older adults at-risk of functional limitations. A comprehensive analysis of the measurement properties of each will be reported. Results will determine the suitability of available assessments and provide guidance to occupational therapists. This information will benefit clients of occupational therapy, occupational therapists and managers in monitoring client outcomes and the effectiveness of interventions.

## Abbreviations

ADL: Activities of daily living; COSMIN: Consensus-based standards for the selection of health measurement instruments; ICF: International Classification of Functioning; MeSH: Medical Subject Headings.

## Competing interests

The authors declare no competing interests.

## Authors’ contributions

KW led the design of the protocol and drafting of the manuscript. LC and NL contributed to the design of the protocol and manuscript revisions. IC assisted in the selection of quality assessments and contributed to manuscript revisions. All authors read and approved the final manuscript.

## Supplementary Material

Additional file 1 OVID Medline Search Strategy (Phase 1). Click here for file
