# Supplementary material for: Functional assessments used by occupational therapists with older adults at risk of activity and participation limitations: a systematic review and evaluation of measurement properties
Source: Syst Rev. 2012 Oct 15;1:45. doi: 10.1186/2046-4053-1-45 (PMC3582570; doi:10.1186/2046-4053-1-45)
Supplement: Additional file 1 — OVID Medline Search Strategy (Phase 1). [file 2046-4053-1-45-S1.docx]

|  | **Medline Search Strategy** |
| --- | --- |
| 1 | randomized controlled trial.pt. |
| 2 | controlled clinical trial.pt. |
| 3 | randomized.ab. |
| 4 | placebo.ab. |
| 5 | drug therapy.fs. |
| 6 | randomly.ab. |
| 7 | trial.ab. |
| 8 | groups.ab. |
| 9 | 1 or 2 or 3 or 4 or 5 or 6 or 7 or 8 |
| 10 | exp animals/ not humans.sh. |
| 11 | 9 not 10 |
| 12 | Occupational Therapy/ |
| 13 | "Activities of Daily Living"/ |
| 14 | exp rehabilitation, vocational/ or rehabilitation/ or self care/ |
| 15 | automobile driving/ or exp transportation/ |
| 16 | "Task performance and analysis"/ or "Time and motion studies"/ or Work Simplification/ |
| 17 | exp leisure activities/ |
| 18 | home care services/ or Home care services, hospital-based/ |
| 19 | Recovery of function/ |
| 20 | exp work/ or human activities/ |
| 21 | social adjustment/ or social behavior/ or social facilitation/ |
| 22 | social environment/ or social support/ |
| 23 | goals/ |
| 24 | occupational therap$.tw. |
| 25 | (activities of daily living or adl$ or eadl$ or iadl$).tw. |
| 26 | rehabilitation.tw. |
| 27 | ((self or personal) adj5 (Care or manage$)).tw. |
| 28 | (Dressing or feeding or eating or toilet$ or bathing or mobil$ or driving or public transport$).tw. |
| 29 | ((daily or domestic or house or home) adj5 (activit$ or task$ or skill$ or chore$)).tw. |
| 30 | leisure.tw. |
| 31 | (recover$ adj5 function$).tw. |
| 32 | self-help devices/ |
| 33 | assistive technology/ or assitive devices/ |
| 34 | (social adj5 (activit$ or function$ or support$ or skill$ or adjust$ or behavio?r or facilitat$)).tw. |
| 35 | or/12-34 |
| 36 | exp "Aged, 80 and over"/ or exp Aged/ |
| 37 | exp Frail Elderly/ |
| 38 | (older or senior$ or elderly or geriatric or frail).tw. |
| 39 | (older adj5 (adult or people or person$)).ti. |
| 40 | (elderly or senior or geriatric or frail or older).ti. |
| 41 | or/36-40 |
| 42 | 11 and 35 and 41 |
| 43 | community health services/ |
| 44 | community hospital/ |
| 45 | ambulatory care/ |
| 46 | outpatient clinics, hospital/ |
| 47 | ambulatory care facilities/ |
| 48 | day care/ |
| 49 | patient care/ |
| 50 | continuity of patient care/ |
| 51 | patient care team/ |
| 52 | patient transfer/ |
| 53 | primary health care/ |
| 54 | comprehensive health care/ |
| 55 | rehabilitation centers/ |
| 56 | community health centers/ |
| 57 | outpatients/ |
| 58 | health services for the aged/ |
| 59 | community.tw. |
| 60 | domiciliary.tw. |
| 61 | hospital$.tw. |
| 62 | early supported discharge.tw. |
| 63 | day?patient$.tw. |
| 64 | outreach.tw. |
| 65 | patient care team.tw. |
| 66 | multidisciplinary team.tw. |
| 67 | health facilities/ |
| 68 | hospitals, convalescent/ |
| 69 | inpatients/ |
| 70 | patients/ |
| 71 | exp hospitals/ |
| 72 | outpatient$.tw. |
| 73 | ((patient$ adj5 discharg$) or (hospital$ adj5 discharg$) or (discharg$ adj5 plan$)).tw. |
| 74 | $patient discharge/ |
| 75 | $patient readmission/ |
| 76 | (readmission$ or re-admission$ or (length adj of adj stay)).tw. |
|  |  |
| 77 | Or43/76 |
| 78 | 42 and 77 |
| 79 | limit 78 to humans |
| 80 | limit 79 to English language |
